# Branching out: New “out of the seed” hypothesis explains increased specialization of young genes during seed maturation

**DOI:** 10.1093/plcell/koaf275

**Published:** 2025-11-13

**Authors:** Julie Robinson

**Affiliations:** Assistant Features Editor, *The Plant Cell*, American Society of Plant Biologists; HudsonAlpha Institute for Biotechnology, Huntsville, AL 35806, USA

All organisms are under constant evolutionary pressure that leads to species-wide changes in gene sequences and expression patterns over time. However, different genes are subjected to different degrees of evolutionary pressure due to various factors. Evolutionarily old genes are those that date back to the common ancestors of multicellular organisms. They tend to be critical for the most basic cellular processes, such as those involved in cell division and protein translation. Changes to highly conserved older genes tend to be lethal because they have become evolutionarily streamlined. On the other hand, relatively newer genes control more specialized functions, such as pathogen resistance or endosperm development. It is thought that newer genes have fewer functional constraints than older genes and thus have more wiggle room when it comes to tolerating changes (Wolf et al. 2009). Newer genes therefore serve as excellent fodder for the continued evolution of new traits and overall plant plasticity.

**Asif Ahmed Sami and colleagues (Sami et al. 2025)** present new work that investigates evolutionary patterns through different stages of seed development in angiosperms. The authors generated transcriptome age index (TAI) profiles using publicly available Arabidopsis RNAseq data from the sequentially occurring embryogenesis, maturation, and germination phases. TAI values convey the evolutionary age of part of a transcriptome, with younger genes being assigned higher values and older genes being assigned lower values (Domazet-Lošo and Tautz 2010). It has previously been shown that an hourglass pattern of TAI values exists in the embryogenesis and germination phases, with older, more highly conserved genes being expressed in the middle of each of these phases (Quint et al. 2012; Drost et al. 2016). In this work, Sami and colleagues report their observation of a “reverse hourglass” pattern of TAI values across Arabidopsis seed development, with significantly elevated TAI values observed during the maturation phase relative to the previous embryogenesis phase and the subsequent germination phase (Figure). This pattern thus indicates increased expression of younger genes during the maturation phase than during the neighboring embryogenesis and germination phases.

**Figure.**
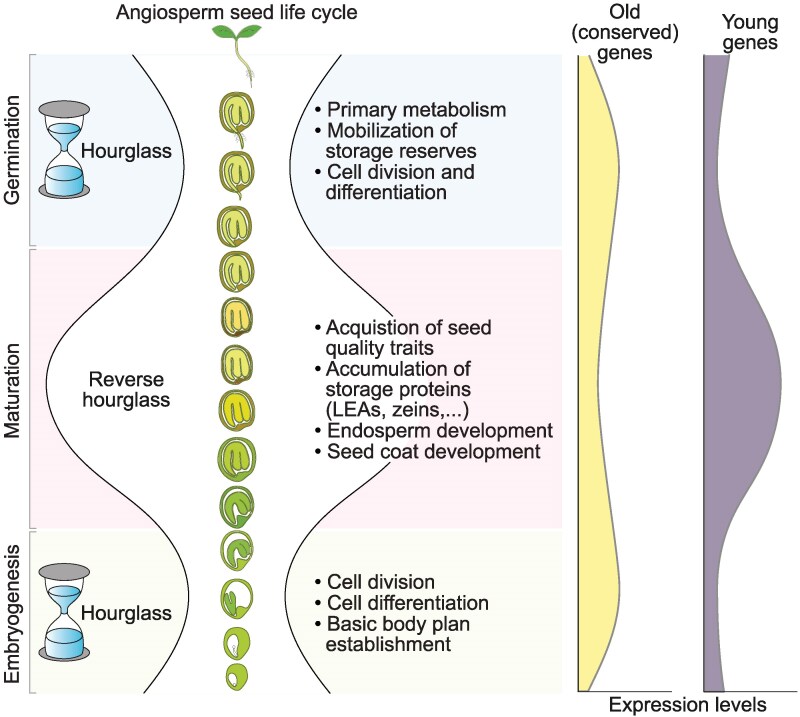
Reverse hourglass of TAI values in angiosperm seed development. The expression of older genes with more foundational functions is emphasized during the embryogenesis and germination phases, while the expression of newer genes with more specialized functions is emphasized during the maturation phase. Reprinted from Sami et al. (2025), Figure 6.

To determine whether the reverse hourglass pattern of TAI values could be a conserved feature of angiosperm seed development, Sami and colleagues performed the same analysis in the eudicots *Brassica napus*, *Solanum lycopersicum* (tomato), and *Glycine max* (soybean) and in the monocot *Zea mays* (maize). Remarkably, they observed the reverse hourglass in all of these species, suggesting that it is indeed conserved in angiosperms. In eudicots, multiple younger gene groups contributed to high maturation TAI; however, in the monocot *Z. mays*, primarily one young gene group (Zein genes) contributed.

Seed maturation is a crucial developmental phase that highly impacts a seed's ability to survive diverse environmental conditions (Gutierrez et al. 2007). Based on their findings, Sami and colleagues propose the “out of the seed” hypothesis that the maturation phase of angiosperm seed development is an evolutionary testing ground for younger genes that fosters evolutionary innovation through the emergence and refinement of more specialized seed functions. The name is a nod to the widely accepted “out of the testis” and “out of the pollen” hypotheses that propose the same idea for these structures (Levine et al. 2006; Wu et al. 2014). When Sami and colleagues compared the top 5% of genes that contribute to increased TAI in the middle of the reverse hourglasses in seeds and pollen, they found that genes unique to each structure were overall younger than those that overlapped. This finding highlights the functional specialization of younger genes in these structures, thus supporting the “out of the seed” hypothesis.

Future studies might look for the presence of the reverse hourglass in other developmental stages and in other plants, including further crop species. By doing so, it may be possible to investigate the effect of domestication on the maturation TAI profiles in cultivated versus wild species and to identify other stages that support functional evolvability in plants.

## Recent related articles in *The Plant Cell*:

- Mariault et al. (2025) investigated the prevalence and evolutionary effects of horizontal gene transfer in plants.
- Robbins and Kelly (2024) dissected the evolution of photosystem subunits in angiosperms at single-residue resolution.
- Chu et al. (2024) used transcriptomics and cistromics to explore variation in gene expression levels in maize.

## Data Availability

No new data were generated or analyzed in support of this article.
